# ATM-Dependent Phosphorylation of All Three Members of the MRN Complex: From Sensor to Adaptor

**DOI:** 10.3390/biom5042877

**Published:** 2015-10-23

**Authors:** Martin F. Lavin, Sergei Kozlov, Magtouf Gatei, Amanda W. Kijas

**Affiliations:** UQ Centre for Clinical Research, The University of Queensland, Brisbane, QLD 4029, Australia; E-Mails: s.kozlov@uq.edu.au (S.K.); m.gatei@uq.edu.au (M.G.); a.kijas@uq.edu.au (A.W.K.)

**Keywords:** DNA double strand breaks, oxidative stress, ATM activation, Mre11/Rad50/Nbs1 complex, phosphorylation, adaptor role

## Abstract

The recognition, signalling and repair of DNA double strand breaks (DSB) involves the participation of a multitude of proteins and post-translational events that ensure maintenance of genome integrity. Amongst the proteins involved are several which when mutated give rise to genetic disorders characterised by chromosomal abnormalities, cancer predisposition, neurodegeneration and other pathologies. ATM (mutated in ataxia-telangiectasia (A-T) and members of the Mre11/Rad50/Nbs1 (MRN complex) play key roles in this process. The MRN complex rapidly recognises and locates to DNA DSB where it acts to recruit and assist in ATM activation. ATM, in the company of several other DNA damage response proteins, in turn phosphorylates all three members of the MRN complex to initiate downstream signalling. While ATM has hundreds of substrates, members of the MRN complex play a pivotal role in mediating the downstream signalling events that give rise to cell cycle control, DNA repair and ultimately cell survival or apoptosis. Here we focus on the interplay between ATM and the MRN complex in initiating signaling of breaks and more specifically on the adaptor role of the MRN complex in mediating ATM signalling to downstream substrates to control different cellular processes.

## 1. Introduction

Exposure of cells to ionizing radiation causes a number of different lesions in DNA including DNA double strand breaks (DSB), the most lethal of these forms of damage. Since DNA DSB represent a major threat to cell viability it is important that they be recognised and repaired timely and accurately. It is also important to bear in mind that this damage is occurring in the context of chromatin where DNA is packaged with histone and non-histone proteins into nucleosomes and higher order structure [1]. It is evident that post-translational modifications of histones and chromatin remodelling factors participate in reorganising chromatin to allow access to DNA signalling and DNA repair proteins [2]. Ionizing radiation-induced damage results in DNA superhelical relaxation which presumably represents the trigger for these changes at the level of chromatin [3]. Poly (ADP-ribosyl)ation of a member of SNF2 chromatin remodelers promotes the sliding of nucleosomes which increases accessibility to DNA to facilitate repair of DNA single strand breaks (SSB) [4,5]. The co-repressor, nucleosome deacetylase and remodelling (NuRD) complex is also recruited to sites of DNA damage. It is suggested that a core component of this complex, CHD4, decondenses chromatin at the damaged site which stimulates ubiquitination by RNF8 and RNF168 to facilitate DNA repair [6]. In addition the epigenetic code on histones is also modified at sites of damage [7].

It seems likely that these changes and others recently described assist in gene silencing in the vicinity of DNA DSB to reduce the risk of further DNA damage as a consequence of the transcription apparatus colliding with the DNA repair complex [8,9]. A number of modifications to chromatin initiate the process of signalling that leads to the accumulation of proteins at the site of damage. Phosphorylation of H2AX on S139 by ATM to form γH2AX is an early event [10]. While phosphorylation of H2AX is dispensable for the initial recognition of DNA DSB this modification is critical for the concentration of several proteins in the vicinity of the damage [11]. These include mediator of DNA damage checkpoint protein 1, (MDC1) which is bound to γH2AX concomitant with dephosphorylation of γH2AX at Y142, a residue that is constitutively phosphorylated on this protein [12,13]. This allows for stabilisation of activated ATM and the MRN complex at the break site to facilitate further H2AX phosphorylation and spreading adjacent to this site [14]. ATM also phosphorylates several other proteins at the site of damage including MDC1, 53BP1, BRCA1, Mre11, Rad50 and Nbs1 [15]. Savic *et al.* [16] have provided evidence that H2AX fuels a γH2AX self-reinforcing mechanism that retains MDC1 and activated ATM on chromatin in the vicinity of the break and that ATM is responsible for promoting γH2AX formation over maximal distances from the break. In the latter case it would be expected that an activated, unbound form of ATM would be responsible for the phosphorylation. However, for the majority of substrates including members of the MRN complex, ATM would be expected to function at or near the site of the DNA DSB.

In addition to phosphorylation, a number of other protein post-translational modifications occur at the break site. Acetylation and deacetylation of histones alters chromatin structure increasing the accessibility of DNA repair proteins. Tip60 acetyltransferase not only alters chromatin structure but once recruited to sites of breaks by the MRN complex binds to H3K9 becoming activated, and in turn acetylates ATM which participates in its activation [17]. Methylation of H3K9 (me3), which gives rise to transient formation of repressive chromatin, is responsible for Tip60 and ATM activation [18]. Histone methylation in response to DNA damage is also implicated in 53BP1 binding [19] and ATM-dependent phosphorylation of the methyltransferase MMSET is required for this accumulation [20]. ATM-dependent phosphorylation of MDC1 also leads to recruitment of RNF8, a ubiquitin ligase [21]. This recruitment occurs through interaction of the FAA domain of RNF8 with phosphorylated MDC1 [22]. RNF8 ubiquitinates histones H2A and H2AX leading to the retention of DNA damage factors at the site of damage to ensure maintenance of genomic integrity. Amplification of ubiquitination by a second ligase, RNF168, which interacts with ubiquitinated H2A in an RNF8-dependent manner, ensures the recruitment and retention of BRCA1, Rap80, Rad18 and 53BP1 at breaks.

## 2. Sensing DNA Double Strand Breaks

Since the DNA DSB represents a potentially lethal lesion it is not surprising that complex recognition and repair mechanisms exist to combat such damage [23]. These breaks are detected and repaired by two major pathways, non-homologous end-joining (NHEJ) which operates throughout the cell cycle but is primarily important in G1 phase [24] and homologous recombination (HR) which relies on the presence of a sister chromatid in late S/G2 phase [25]. Breaks are also repaired by microhomology-mediated end-joining (MHEJ) and single strand annealing (SSA) pathways [26]. Ku70 and Ku80 are the essential sensors for DNA free ends in repair by NHEJ [27]. Once bound to DNA ends these proteins recruit DNA-PKcs, the XRCC4/Ligase 4 heterodimer, XLF and PAXX protein to complete the process of repair [28]. While HR operates later in the cell cycle than NHEJ there are mechanisms in place that regulate pathway choice. DNA-PKcs antagonises DNA DSB resection by blocking the recruitment of the resection nuclease, Exo1, while the MRN complex stimulates resection in the presence of Ku and DNA-PKcs, enhancing autophosphorylation of DNA-PKcs and inhibiting XRCC4/DNA ligase end-joining [29]. The single strand DNA binding protein (SSB1) binds directly to Nbs1 and stimulates the endo-exonuclease activity of the MRN complex favouring HR [30]. In addition, CDK targeting of Nbs1 promotes DNA-end resection, replication restart and HR [31]. Other factors that influence pathway choice include modifications to histone proteins [32]; sumoylation of the end resection protein Sae2 [33]; ATM-mediated MOF phosphorylation [34] and by the opposing activities of 53BP1 and BRCA1 [35]. These are just some examples of proteins and post-translational events that influence pathway choice.

**Figure 1:**
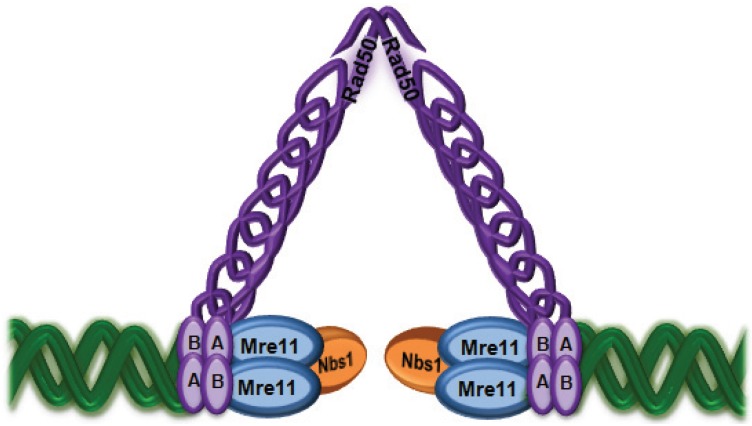
The MRN complex tethers DNA broken ends. The Mre11/Rad50 complex binds to DNA ends as a heterotetramer through binding sites on Mre11. Bringing the ends together is achieved by Rad50 globular domains (A and B) and bridging of the DNA molecules by the Zn-hooks. Nbs1 is required for nuclear localisation of Mre11/Rad50 and regulates their activities.

The MRN complex is the other major sensor of DNA DSB. It has been implicated in both major mechanisms of DNA DSB repair, DNA replication fork restart, telomere maintenance, meiosis and signalling to the cell cycle checkpoints [36,37]. Evidence of a role for the MRN complex members in the DNA damage response (DDR) comes from the detection of patients with mutations in these genes [38,39,40]. Patient cells were characterised by chromosomal instability, radiosensitivity, radioresistant DNA synthesis, and cell cycle defects [37]. The major characteristics of these disorders appear in Table 1. Structural representations of the MRN complex depict Rad50 proteins with interacting globular domains (Walker A and B motifs) from which anti-parallel coiled-coil structures extend to Zn-hook domains that enable Rad50 dimerization [41]. The ATP-binding globular domains interact with 2 molecules of Mre11 and it seems likely that both molecules are involved in DNA binding followed by recruitment of Nbs1 which provides for interaction with ATM for activation of signalling (Figure 1). Rad50 promotes long-range allosteric effects through the coiled-coil and Zn-hook domains and its ATPase activity is responsible for transitions between monomeric and dimeric forms that impact on resection and rejoining activities [42]. Use of crystal structures, X-ray scattering and biochemical and functional analysis of mutant Rad50 complexes demonstrate that the ATP-bound closed conformation promotes DNA end-binding and end-tethering whereas ATP hydrolysis is essential for end-resection [43]. This switch from tethering to resection appears to be responsible for activation of the nuclease and resection activity of Mre11, independent of capacity to activate ATM [44]. It seems likely that the ATP-bound form of MRN is the critical conformation required for ATM activation [45].

**Table 1:** Characteristics of DNA damage recognition disorders.

Genetic disorder	Abbreviation	Gene/Protein	Neurological features	Cancer pre-disposition	Chromosomal instability	Radio-sensitivity	Cell cycle checkpoint defect	DNA DSB repair defect
Nijmegen breakage syndrome	NBS	NBN/Nbs1	Microcephaly; Mental retardation	Yes	Yes	++++	Yes	Yes
Nijmegen breakage-like disorder	NBLD	Rad50	Microcephaly; Mental retardation	?	Yes	++	Yes	Yes
Ataxia-telangiectasia-like disorder	ATLD	Mre11	Cerebellar ataxia; Ocular apraxia; microcephaly (2 patients)	Yes	Yes	++	Yes	Yes
Ataxia telangiectasia	A-T	ATM	Cerebellar ataxia; Ocular apraxia	Yes	Yes	++++	Yes	Yes

## 3. Importance of the MRN Complex in ATM Activation

ATM is a central player in the DDR acting to initiate signalling to a number of cellular processes including cell cycle control, transcription and DNA repair [46]. This protein is mutated in ataxia-telangiectasia (A-T) an autosomal recessive disorder characterised by immunodeficiency, cancer predisposition, neurodegeneration, chromosomal instability, radiosensitivity and defective cell cycle checkpoint activation [15,47]. These characteristics overlap with those due to mutations in NBN (Nijmegen Breakage Syndrome), Mre11 (ATLD) and Rad50 (Nijmegen breakage-like disorder) (See Table 1).

**Figure 2:**
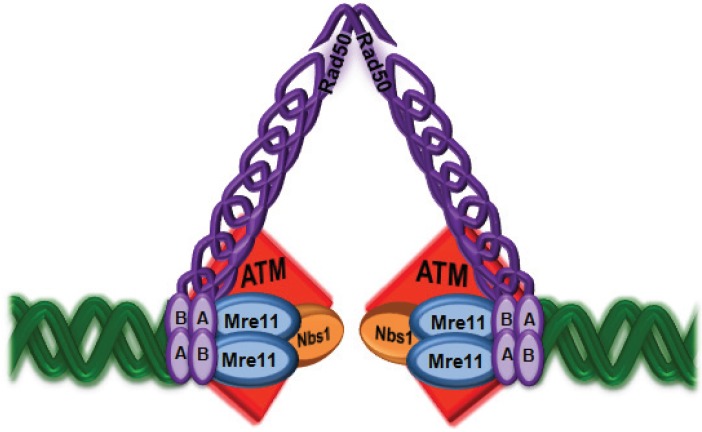
ATM is recruited to DNA ends by the MRN complex. Once localized to the site of damage ATM is activated by monomerization and autophosphorylation. In this figure ATM is depicted as two monomers bound to each side of the break through their interaction with Nbs1.

The similarities in clinical and cellular features between A-T and disorders of the MRN complex pointed to an overlapping role in dealing with DNA damage. Mirzoeva and Petrini [48] observed rapid nuclear retention of the MRN complex in small granular foci in response to radiation exposure and this was not dependent on ATM kinase supporting a role for the complex in recruiting ATM to the site of damage (Figure 2). Uziel *et al.* [49] subsequently showed that ATM activation was defective in cells from patients with MRN deficiencies. Dose response and time course experiments indicated that ATM activation was reduced in NBS cells, and most severely affected in a form of ATLD (S) homozygous for a nonsense Mre11 mutation. These data suggested that the MRN complex was necessary but not essential for ATM activation. Other data that support such a role include infection with an adenovirus lacking the E4 region that induces a DNA damage response with ATM activation [50]. Under these conditions wild type adenovirus blocks this signalling by degrading the MRN complex.

ATM is predominantly a nuclear protein but there is some evidence for its presence in the cytoplasm where it has been shown to be associated with both mitochondria and peroxisomes [51,52]. Its presence in the cytoplasm would suggest that it is more likely to be activated by a mechanism not involving DNA damage which may be more relevant to its role in post-mitotic cells (discussed below) [46]. ATM is normally present in the nucleus as an inactive dimer which monomerizes in response to DNA DSB [53]. This monomerization is associated with autophosphorylation on S1981 which is thought to be the signal for ATM activation. However, it is evident that ATM is autophosphorylated at multiple sites, in response to DNA breaks, including S367, S1893, S2996 and T1885 [54,55]. It seems likely that these sites are functionally important since mutants for these sites fail to restore ATM signalling in A-T cells. The importance of these sites for ATM activation in mice is less clear since mutations that disrupt one or more of these sites in murine Atm do not interfere with ATM signalling or function [56,57]. Use of an *in vitro* reconstituted system for ATM activation and *Xenopus* extracts with high concentrations of DNA ends also failed to show a requirement for ATM autophosphorylation [58,59]. Under these conditions amount of DNA ends alone was sufficient to monomerise the dimer and activate ATM in the absence of the MRN complex. At lower concentrations of DNA ends this did not occur. These data suggest that the MRN complex may be unmasking a DNA-binding region on ATM to facilitate activation [46]. Acetylation of ATM at K3016 by the histone acetyltransferase Tip60 is also a key step linking the detection of DNA damage to the activation of ATM [60]. This is achieved by tyrosine phosphorylation of Tip60 in response to DNA damage which promotes its binding to H3K9me3 and subsequent acetylation and activation of ATM [61]. It is also evident that ATM is activated by gross changes in chromatin structure [53] and by RNF8 and CHFR-mediated chromatin relaxation by histone ubiquitination [62]. More recently it has been demonstrated that ATM is activated by R-loops (RNA/DNA hybrids) at transcription blocking lesions to impede spliceosome organization and augment alternative splicing triggered by DNA damage [63]. This was observed in the absence of DNA DSB.

## 4. ATM Activation by Oxidative Stress

Oxidative stress has been implicated both in A-T patients and in the cellular phenotype [64,65,66]. Shortly after ATM was cloned [67], it was suggested that this protein acted as a sensor of reactive oxygen species and/or oxidative damage to cellular macromolecules [68]. It was envisaged that ATM induced signalling through multiple pathways, coordinating acute phase cell responses with cell cycle control and DNA repair. While there is mounting evidence for the involvement of oxidative stress in the A-T cellular phenotype and while it may well contribute to the pathogenesis in patients there is little direct evidence to support this. Short-term treatment of A-T patients with oral or intra-erythrocyte infusion of steroids ameliorates the neurological signs [69,70,71,72]. Improvement was particularly evident in speech disturbance, stance and finger pointing criteria. The basis for protection by betamethasone remains unresolved but it has been suggested that its anti-inflammatory action as a steroid may be responsible. This is in keeping with an oxidative stress phenotype which may contribute to the inflammatory changes by increasing cytokine levels. Indeed there is support for this from the observation that IL-8 levels are significantly higher in the serum of A-T patients [73]. Evidence that inflammation contributes to the pathogenesis in A-T is accumulating. The association of accelerated aging, development of cardiovascular disease and insulin resistance with A-T provides a link to inflammation [74]. Furthermore, unrepaired DNA present in the cytoplasm of A-T cells and in Atm^−/−^ mice primes the type 1 IFN system through the STING pathway to promote an innate immune response [75,76]. It seems likely that defects in innate immunity may contribute to various aspects of the A-T phenotype including neuro-degeneration.

While the Atm mutant mouse does not exhibit neurodegeneration there is stronger evidence for an association with oxidative stress. Barlow *et al.* [77] were the first to show that organs which develop pathological changes in ATM-deficient mice are targets for oxidative damage, particularly Purkinje cells. At the mRNA level haemoxygenases (HO-1 and HO-2) were elevated in the cerebellum and to a lesser extent in the cerebral cortex in Atm^−/−^ mice. This elevation was also evident at the protein level for HO-1 in the cell soma and dendrites of Purkinje cells. While these cells exhibit oxidative stress there was no evidence of apoptosis or neurodegeneration in these animals. Subsequent studies revealed reduced *in vitro* survival of Purkinje neurons from Atm mutant mice and reduced dendritic branching in these cells [78]. Use of an antioxidant (isoindoline nitroxide) prevented this Purkinje cell death, enhanced dendritogenesis and when fed to pregnant mice had a small enhancing effect on Purkinje cell survival. Antioxidants have also been shown to increase the lifespan of Atm^−/−^ mice by delaying the onset of thymic lymphomas and also corrected neurobehavioural deficits in these mice [79,80,81]. Upregulation of AMP-activated protein kinase (AMPK) and elevation of ROS occur coordinately in the cerebella of Atm^−/−^ mice and administration of the antioxidant monosodium luminol to these mice attenuates this upregulation and corrects neuromotor deficits [82]. Finally Quick and Dugan [83] found increased levels of ROS in the cerebellum and striatum but not the cortex of Atm^−/−^ compared to Atm^+/+^ mice. Confocal microscopy revealed elevated superoxide levels in cerebellar Purkinje cells and nigral dopamingergic neurons but not in cortical neurons. 

As indicated above ATM has been shown initially to respond to DNA DSB but recent data provide evidence that ATM is activated by DNA SSB and coordinates their repair with DNA replication [84]. There is also evidence that ATM is activated by oxidative stress since A-T fibroblasts, unlike controls, treated with t-butyl hydroperoxide failed to show activation of cell cycle checkpoints and signalling to downstream substrates [85]. More recently Paull and her colleagues have provided important new information on the mechanism of ATM activation by oxidative damage and showed that it differed from that initiated by DNA DSB. In short, they showed that oxidation of Atm directly induced Atm activation in the absence of DNA DSB and was not dependent on the presence of the MRN complex [86]. They showed that the oxidized form of ATM was a disulphide cross-linked dimer and mutation in C2991, a critical cysteine residue, blocked ATM activation through this pathway. While DNA DSB activate ATM by monomerization of an inactive dimer, activation by oxidative stress leads to dimerization of ATM via a disulphide bond. Thus ATM is acting as a redox sensor forming a disulphide bond which leads to modulation of its activity which has also been observed for other proteins either activated or inactivated by induced disulphide bond formation [87,88]. Unlike that observed for ATM responding to DNA DSB, where it is localized to sites of DNA damage in discrete foci, ATM activated by oxidative damage is diffusely distributed throughout the nucleus and activation is prevented by the antioxidant, *N*-acetylcysteine [89]. It is also of interest that resveratrol stimulates ATM activation, as determined by autophosphorylation and substrate phosphorylation in a manner promoted by ROS [90]. Agents that prevent disulphide bond formation substantially reduced its effect on ATM activation. Overall it is evident that ATM can be activated by agents that do not change DNA under the conditions employed. At this stage the data do not reveal that the ATM activated by oxidative stress is nuclear, cytoplasmic or both. However, since ATM has been detected in the cytoplasm at least in some cell types including Purkinje cells it will be of interest to determine whether this form is activated. In this context it is notable that at least some of the cytoplasmic ATM is associated with mitochondria and peroxisome organelles where ROS are generated [51,52]. There is also evidence for the presence of a nuclear localisation signal within the amino terminus of ATM which could affect its distribution between the nucleus and cytoplasm [91]. It is therefore tempting to suggest that ATM acts as a sensor of ROS in these organelles and responds by phosphorylating specific substrates that function in the stress response. At this stage there is limited information available on ROS-activated ATM substrates. ROS activated ATM, in the absence of DNA damage, is diffusively spread throughout the nucleus and leads to phosphorylation of p53 and Chk2 but not Kap-1 [89]. Diffuse spreading of activated ATM in the nucleus is also observed after ATM activation by hypoxia [92]. However, under these conditions Kap-1 phosphorylation was observed adding to the complexity. These data point to activation of nuclear ATM by H_2_O_2_ but do not show that the cytoplasmic form is being activated. To address this it is necessary to identify cytoplasmic substrates for ATM. A recent report provides data supporting such a role for ATM at the peroxisome [93], they showed that ATM is localised to the peroxisome by binding to the import receptor PEX5. In response to ROS ATM phosphorylated PEX5 at S141, promoting PEX5 monoubiquitilation at K209, which directs the autophagosome to peroxisomes to induce pexophagy.

**Figure 3:**
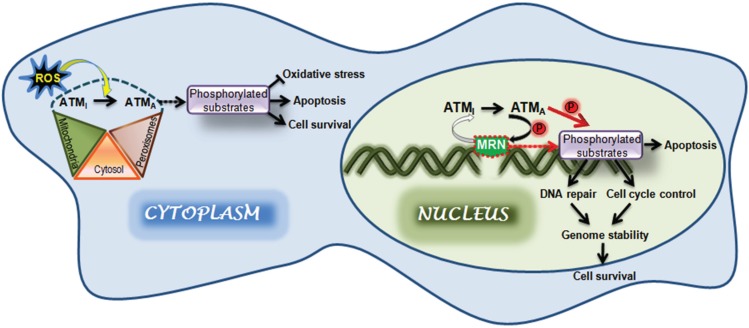
ATM activation in the nucleus and cytoplasm. ATM is predominantly localized to the nucleus where it is recruited to DNA DSB by the MRN complex and activated. Upon activation it phosphorylates a number of substrates including members of the MRN complex. These phosphorylated proteins then act in signalling pathways to participate in DNA repair, cell cycle control and induction of apoptosis. These events combine to protect the stability of the genome, maintain cell survival and minimize the risk of pathological change. ATM is also activated by oxidative stress under conditions that do not inflict DNA damage. Here we show activation of ATM in the cytoplasm by ROS but clearly this may also occur in the nucleus. ATM is present in the cytoplasm and has been shown to be localized to both peroxisomes and mitochondria. It is not known at this stage how ATM from these different cytoplasmic compartments responds to oxidative damage. Furthermore, there is no compelling evidence that cytoplasmic ATM phosphorylates specific substrates but is envisaged that such phosphorylations is part of the response to oxidative stress.

While there are many candidate substrates for ATM in the nucleus few have been identified in the cytoplasm except in large-scale phosphoproteomic analysis without subsequent validation [94,95,96]. Li *et al.* [97] selected two vesicle proteins from these lists, VAMP2 and synapsin-I, proteins that function during neurotransmitter release. They demonstrated physical association between ATM, VAMP2 and synapsin-I but it was not clear whether either of these proteins was a substrate for ATM and under the conditions employed ATM was not autophosphorylated, suggesting it was not activated in cerebellar extracts. Another possibility is the microtubule-associated protein, NuSAP, which interacts with ATM and is phosphorylated by ATM on S124 in the G2/M phase of the cell cycle to activate the checkpoint [98]. Since this phosphorylation occurs during mitosis it is difficult to determine the source of ATM. Should this protein perform a DNA damage independent role in the brain, where it has been shown to be at least partially distributed in the cytoplasm, it is important to show that the cytoplasmic form can be activated and also to identify substrates in order to understand the role of ATM in the brain. Figure 3 depicts the activation of ATM both in the nucleus and cytoplasm. ATM is activated in the nucleus by DNA DSB which is dependent on MRN whereas in the cytoplasm ATM is activated by ROS independent of the presence of MRN and DNA DSB. These data would suggest that under these conditions members of the MRN complex would not be phosphorylated by ATM since recognition of DNA DSB is not involved. Furthermore it has been demonstrated that when ATM is activated by low levels of ROS a full DNA damage response does not occur as determined by substrate phosphorylation [46].

## 5. Functional role of MRN Complex Members as Substrates for ATM

ATM is one of several members of the PI3K-like protein kinases (PIKKs) that respond to DNA damage and other cellular stresses by phosphorylation of a range of substrates [46,99]. Others include ATM and Rad3-related protein, ATR, the catalytic subunit of DNA-dependent protein kinase DNA-PKs; suppressor of morphogenesis in genitalia-1(SMG-1) and mammalian target of rapamycin (mTOR) kinases. DNA-PK plays a central role in non-homologous end joining (NHEJ) of DNA DSB largely during the G1 phase of the cell cycle as well as a V(D) J recombination [100,101]. ATR responds to single strand DNA primarily at sites of DNA replication, fork blockage and collapse [102]. SMG-1 phosphorylates Upf1 in mammalian nonsense-mediated decay [103], localises to stress granules [104] and plays a role as a tumour suppressor [105]. mTOR, made up of the distinct complexes TORC1 and TORC2, functions as a nutrient sensor by regulating availability of cellular energy substrates required for cell growth [106]. All of these PIKK family members phosphorylate a multitude of substrates in their roles in controlling different cellular processes. While these protein kinases are activated by different stimuli it is also evident that overlap in their signalling pathways occur in that they give rise to phosphorylation of the same substrates [99]. ATM plays a central role in phosphorylating hundreds of proteins in response to DNA DSB which not surprisingly are enriched for proteins involved in the DDR [94]. Many of these are phosphorylated on consensus sites (S/TQ) but it is also evident that these proteins are phosphorylated on non-consensus sites which could be due to direct ATM phosphorylation or by another kinase in an ATM-dependent manner [99]. What emerges is the identification of a large number of protein networks not previously linked to the DDR and controlling a variety of cellular processes [94]. As described above it is evident that the MRN complex plays a key role in recruitment and activation of ATM at the site of damage for this signalling. What is less articulated is the unique role of this complex in mediating that signalling not only by its physical presence at the sites of damage but also by post-translational modification of the individual proteins in the complex by phosphorylation.

### 5.1. ATM-Dependent Signalling by Nbs1 Phosphorylation

**Figure 4:**
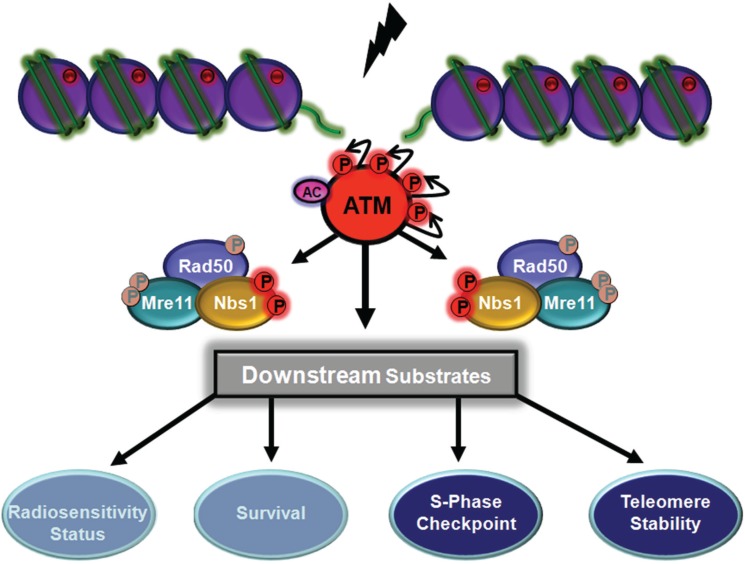
ATM-dependent phosphorylation of Nbs1 and downstream signalling. Nbs1 is phosphorylated on two sites (S278, S343) in response to DNA DSB. Once phosphorylated, Nbs1 acts as an adaptor in mediating control of the S phase checkpoint and in telomere maintenance. There is also evidence, for and against, that these phosphorylations impact on radiosensitivity and cell survival (shaded in figure). The phosphorylations on Rad50 and Mre11 are shaded in this figure to reveal emphasis on Nbs1 phosphorylation but it is clear that these are occurring simultaneously. Post-translational modifications to ATM are also shown.

A conserved carboxy-terminal motif in Nbs1 is required for its interaction with ATM and for both the recruitment and activation of ATM for downstream signalling [107,108]. Once activated ATM phosphorylates a number of substrates including all 3 members of the MRN complex. These phosphorylations activate downstream protein kinase, alter protein-protein interactions, modify the capacity of proteins to bind to chromatin and alter chromatin structure to regulate various cellular processes. However, it is evident that phosphorylation of members of the MRN complex is responsible for the mediation of signalling events by their capacity to regulate phosphorylation of downstream substrates, functionally important in DNA repair, cell cycle control and cell survival [109]. It is also evident that phosphorylation of the individual members of the complex exerts different effects on cellular processes. Nbs1 is phosphorylated by ATM on two sites S278 and S343 in response to radiation damage [110]. A construct mutated in the S343 phosphorylation site abrogated the S phase checkpoint induced by radiation in control cells but failed to compensate for this functional deficiency in NBS cells [111]. Interruption of the S343 phosphorylation site has also been shown to compromise survival and radiosensitivity after DNA damage but not affect the stability of the MRN complex or capacity to form foci [112,113]. On the other hand Zhao *et al.* [110] found that phosphorylation at S278 and S343 was essential for the cellular response to DNA damage including S phase checkpoint activation, formation of nuclear foci and rescue of hypersensitivity to ionising radiation. In contrast to that report a later study employing expression of NBN, containing mutations in the ATM-targeted phosphorylation sites (S278,S343), did not resolve S phase checkpoint control but did resolve the ability of radiation to activate Chk2, induce nuclear foci formation and normalize radiosensitivity in NBS cells [114]. However expression of NBN containing mutations in forkhead-associated or BRCT domains did not correct radiosensitivity but did resolve the S phase checkpoint in NBS cells. Generation of a “knockin” mouse with a point mutation in S278(S278A) demonstrated that phosphorylation at this site was dispensable for mouse development but showed radiation dose dependency in mediating signalling through Chk2 and SMC1 [115]. Radiation-induced phosphorylation of Chk2 and inhibition of the mitosis-inducing phosphatase cdc25C by Chk2 phosphorylation prevents the passage of cells into mitosis [116]. This signalling pathway is defective in NBS and can be corrected by introduction of wild-type Nbs1 but not by the S343A phosphorylation site mutant [117]. This report provides evidence that Nbs1 (phosphorylated) acts as an adaptor for signalling through Chk2 and cdc25C to the G2/M checkpoint. Structural maintenance of chromosome protein 1 (SMC1) is a component of the DDR network that functions as an effector in the ATM/Nbs1-dependent S phase checkpoint pathway [118]. Activation of this checkpoint requires ATM-dependent phosphorylation of SMC1 on S957 and S966. These phosphorylations are, in turn dependent on ATM-dependent phosphorylation of Nbs1 at S278 and S343. These data point to an adaptor role of phosphorylated Nbs1 in an ATM/Nbs1/SMC1 pathway that signals to the S phase checkpoint (Figure 4). This appears to be separate and parallel to a second pathway, ATM/Chk2/cdc25A, that also regulates the S phase checkpoint pathway [118]. ATM-dependent phosphorylation of SMC3 has also been shown to be important for the S phase checkpoint [119]. The MRN complex is also involved in telomere maintenance since shortened telomeres have been observed in NBS cells [120,121,122]. Inducible expression of Nbs1 (S278A/S343A) in human tumour cells led to an increased rate of telomere loss compared to wild-type, indicating that ATM-dependent phosphorylation of Nbs1 is important for telomere maintenance [120]. Loss of telomeres did not correlate with radiosensitivity or radioresistant DNA synthesis. It is not clear from this study how phosphorylation of Nbs1 might function in sustaining the telomere. It is evident from these reports that Nbs1 phosphorylation, at least on 2 sites, plays a role in downstream signalling through other phosphorylated proteins (e.g., pChk2 and pSMC1) to regulate cellular events. There are also discrepancies and contraindications between the different reports as to the influence of such phosphorylations on these events, however, there appears to be consensus on the finding that Nbs1 phosphorylation is critical for control of the S phase checkpoint (Figure 4). What is not clear is why Nbs1 phosphorylation is required for this checkpoint but not for the others defective in NBS cells [123].

### 5.2. ATM-Dependent Signalling Through Rad50

**Figure 5:**
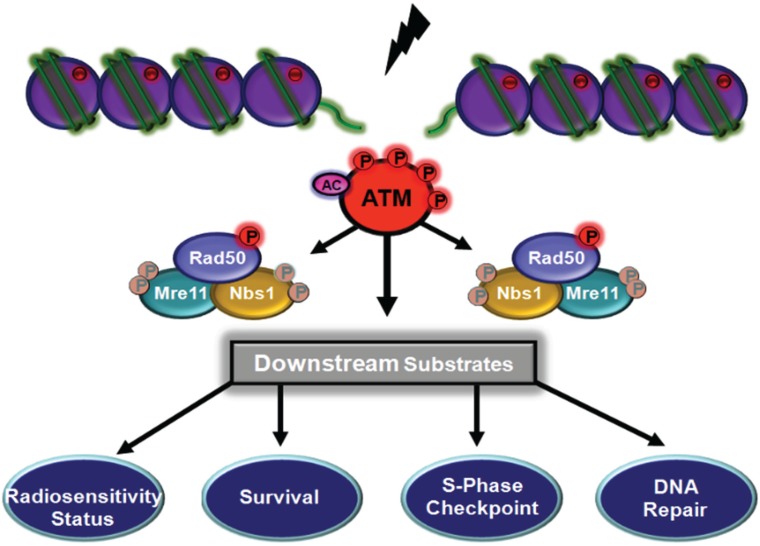
ATM-dependent phosphorylation of Rad50. Rad50 is phosphorylated at a single site (S635) in response to DNA DSB. This protein also acts in an adaptor role, once phosphorylated, to signal not only to the S phase checkpoint but also to mediate DNA repair. Phosphorylation at this site is also important in protecting against radiation damage and for survival of the cell. Shading of Nbs1 and Mre11 phosphorylations as described in Figure 4.

To date only a single patient has been identified with Rad50 deficiency. This patient was initially diagnosed with microcephaly, mental retardation, a bird-like face and small stature and thought to be an NBS patient [124]. However, it was subsequently shown that this patient was a compound heterozygote for mutations in Rad50 that gave rise to low levels of an unstable Rad50 protein [38]. Patient cells were characterised by radiosensitivity, chromosomal instability, failure to form MRN foci, impaired cell cycle checkpoints and defective ATM-dependent signalling post-irradiation. As part of the MRN complex, Rad50 promotes long-range allosteric interactions through the coiled-coil domain and the zinc hook [42]. It’s ATPase activity drives dynamic transitions between dimeric and monomeric forms to control end joining and resection activities. It seems likely that its interaction with Mre11 is important in maintaining the coiled-coil structural features and homodimerization required for DNA tethering [125]. Furthermore, the dimerization state of the Rad50 hook domain is manifested in part by structural modulation of the globular domain, suggesting that transitions between the open and closed forms of the MRN complex are interdependent with transitions in hook-mediated dimerization [41]. Other influences such as post-translational modifications to Rad50 might also be expected to affect its function. Large scale proteomic analysis of proteins phosphorylated in response to DNA damage on consensus sites for ATM and ATR identified more than 900 sites on over 700 proteins including Rad50 [94]. Use of NetworKin database (http://networkin.info) which integrates consensus substrate motifs with context modelling for prediction of cellular kinase substrate relations also identified Rad50 as a phosphorylated substrate [126]. In the latter study they confirmed that Rad50 phosphorylation was ATM-dependent using A-T fibroblasts and Atm^−/−^ cells using a general anti-phospho-S/TQ antibody. Evidence for a specific single site of phosphorylation on Rad50, S635, was subsequently demonstrated in response to DNA DSB [127]. No other sites of phosphorylation on this protein have been reported in response to DNA damage. Phosphorylation did not disrupt the MRN complex and was shown to be functionally important. A phospho-site mutant (S635G) failed to correct the S phase checkpoint in Rad50-deficient cells and also failed to correct radiosensitivity in these cells [127]. Failure of the phospho-site mutant to correct radiosensitivity was consistent with a reduced capacity for repair of DNA DSB in Rad50-deficient cells. The mutant was defective in restoring NHEJ and HR defects in these cells. Previous data had shown that the MRN complex had a role in both NHEJ and HR of DNA DSB [128,129], but this was the first time that phosphorylation of a member of the complexes was shown to be involved. While some reports suggested that Nbs1 phosphorylation influences radiosensitivity the consensus is that this is not the case (see Section 5.1). This specific phosphorylation of Rad50 appears to mediate signalling to both the S phase checkpoint and DNA repair to enhance cell survival post-irradiation (Figure 5).

Evidence was also provided that this signalling was mediated through ATM-dependent phosphorylation of SMC1. Introduction of the Rad50 S635G mutant into the Rad50-deficient cells failed to reduce SMC1 phosphorylation in response to DNA damage. Previous data have revealed that phosphorylation of Nbs1 by ATM is required for SMC1 phosphorylation with Nbs1 acting as an adaptor in the ATM/Nbs1/SMC1 pathway for S phase checkpoint activation [118]. The additional capacity of phosphorylated Rad50 to signal to DNA repair suggests that it is signalling through another substrate. This substrate has not been identified but it is not p53, Chk2 or Kap-1, since signalling through these substrates was normal in phospho-site mutant transfected Rad50-deficient cells.

### 5.3. ATR-Dependent Signalling Through Rad50

**Figure 6:**
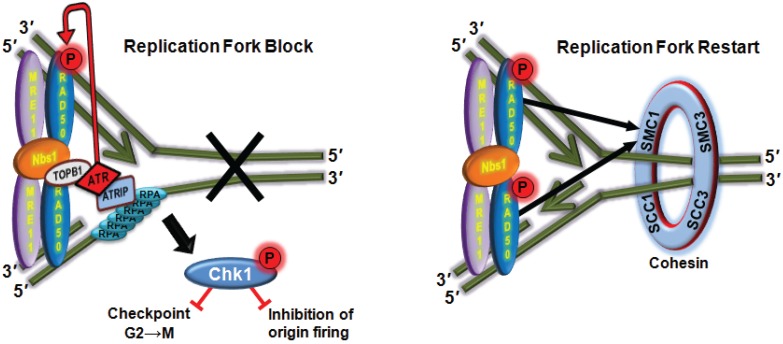
ATM-dependent phosphorylation of Rad50 promotes ATR downstream signalling and DNA replication fork restart. DNA replication stress leads to ATR-dependent phosphorylation of Rad50 on the same site (S635) as induced by ATM after DNA DSB. Rad50 is initially required for ATR activation and in turn is phosphorylated by ATR. The phosphorylated form of Rad50 is essential for DNA replication restart by promoting loading of cohesion at site of damage. Phosphorylation of Rad50 is also required for cell cycle control and survival by signalling through Chk1.

While the MRN complex has key roles in sensing DNA DSB, activating ATM and processing DNA ends its role in ATR activation and signalling is less clear. Several reports provide evidence for and against the involvement of individual members of the complex (Nbs1 and Mre11) in ATR checkpoint activation and signalling [130,131,132]. More recent data using *Xenopus* extracts showed that the MRN complex binds specifically to an ATR-activating structure (ssDNA and a ssDNA/dsDNA junction) and is required for ATR-dependent checkpoint activation by recruitment of the ATR activating factor TopBP1 [133]. These data point to a direct role for MRN in DNA damage recognition and ATR activation at sites of DNA replication blockage and collapse. It was subsequently shown that recruitment of ATR-ATRIP to sites of DNA replication stress and its activation were defective in the absence of Rad50 [134]. Overall the results suggest that the MRN complex mediates ATR signalling in response to replication stress but there is less data on the involvement of phosphorylation of members of the complex in this signalling. One report showed that hydroxyurea (HU)-induced RPA phosphorylation required both the Nbs1 protein and its phosphorylation providing indirect support for its involvement in ATR signalling [135]. Support for a more direct role of phosphorylation of one member of this complex, Rad50, was recently provided. Gatei, *et al.* [134] revealed that Rad50 was phosphorylated after exposure to cells to UV or HU, agents that block replication, on the same site (S635) as observed after IR. This phosphorylation was not detected in Rad50-deficient cells and ATR was responsible. Furthermore, it was demonstrated that not only was it necessary for Rad50 to be recruited to sites of stalled replication forks to initiate ATR-dependent signalling but it also needed to be phosphorylated for this to occur (Figure 6). They also showed that Rad50 phosphorylation was required for restart of DNA replication forks after DNA damage by promoting the loading of the cohesion complex at these sites. Gatei, *et al.* [134] also demonstrated that replication stress-induced Rad50 phosphorylation is functionally significant for cell survival and cell cycle checkpoint activation. Here again is an example of the importance of an adaptor role for a member of the MRN complex, this time in ATR-mediated signalling in response to replicative stress.

### 5.4. ATM-Dependent Phosphorylation of Mre11 Controls Resection

**Figure 7:**
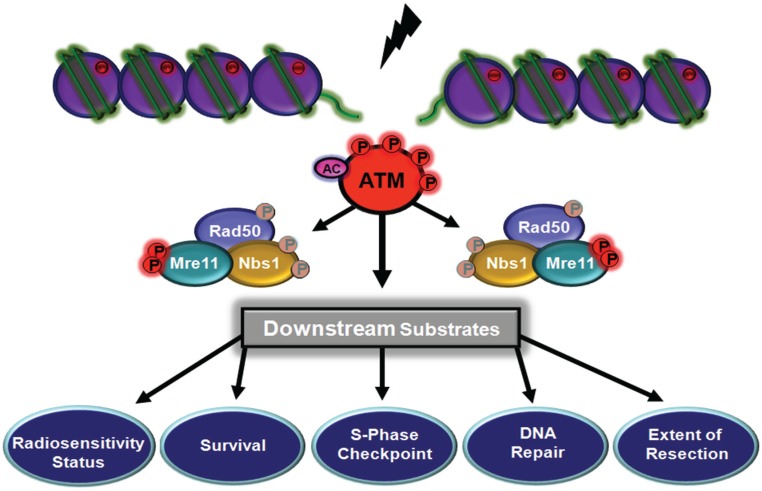
ATM-dependent phosphorylation of Mre11. In response to DNA DSB Mre11 is phosphorylated at adjacent sites (S676, S678). These phosphorylations are important for cell cycle control and cell survival. It seems likely that Mre11 phosphorylations control the extent of end resection and thus DNA repair.

A conserved region within the carboxy terminal sequence of Nbs1 is responsible for its interaction with Mre11 [136]. This interaction localises Mre11 to the nucleus in response to DNA damage. The presence of five conserved phosphoesterase motifs in the N-terminal half of Mre11 are required for its Mn^+2^-dependent 3'→5' exonuclease and ssDNA endonuclease activities [137]. Interaction with the Rad50 coiled-coil base occurs through two α-helicases on Mre11 C-terminal to the nuclease core domain [138]. Mre11 stabilizes dimerization of Rad50 and stimulates its ATPase activity leading to conformational change of both proteins, exposure of the nuclease site and DNA resection [139]. Like the other two members of the complex Mre11 is also phosphorylated but at multiple sites including S676, S678 and S681 [94,126,140]. Evidence for ATM-dependent phosphorylation of Mre11 has also been provided by gel mobility shift after DNA damage, but the sites of phosphorylation were not identified [141,142]. Use of *Xenopus* extracts narrowed these phosphorylation sites to a small region of ATM consensus sites (S/TQ) within the C-terminus of Mre11 [143]. They also demonstrated that hyperphosphorylation of Mre11 inactivated the MRN complex, through dissociation from chromatin, enabling downregulation of DNA damage signalling during cell cycle checkpoint recovery following DNA repair. More recently exposure of cells to IR showed that Mre11 is phosphorylated by ATM at two adjacent sites S676QS678Q [144]. Phosphorylation was not observed after exposure of cells to agents causing cross-links (cisplatin) or DNA alkylation (MMS). Constructs expressing wild-type Mre11 or a phospho-site mutant (MRE11S676AS678A) restored ATM-dependent signalling through the downstream substrates Kap-1 and SMC1. This is quite different to that for Rad50 and Nbs1 phospho-site mutants where correction of signalling was not observed in cell lines deficient for these proteins [127]. A functional role for Mre11 in maintaining cell survival was established by showing that the phospho-site mutant failed to restore a normal pattern of cell survival in ATLD cells (Figure 7). A GFP-based DNA repair reporter assay revealed a defect in both microhomology-mediated NHEJ and HR in Mre11S67A6S678A transfected ATLD cells. Impaired recruitment of Rad51 foci as well as reduced rate of loss of these foci in mutant transfected cells supported the defect in HR. Mre11 has been shown to play a catalytic role during homology directed repair where it nicks the DNA upstream from the break then resects in a 3'→5' direction towards the break with more extensive resection being performed primarily by the 5'→3' Exo1 [145,146]. Analysis of markers for single strand DNA generated during G2 phase showed that Mre11 appeared to be involved in controlling the extent of resection at any given site undergoing homology directed repair.

Phosphorylation of Exo1 is involved in a negative feedback loop to limit accumulation of ssDNA during resection and DNA damage checkpoint activation [147]. In addition ATM-dependent phosphorylation of this protein modulates HR repair of DNA DSB [148]. Immunostaining with an antibody against Exo1 pS714 showed co-localization with γH2AX foci in wild-type Mre11 corrected ATLD cells, but this was markedly reduced in cells transfected with Mre11S676AS678A mutant transfected cells [144]. The reduced phosphorylation of Exo1 was consistent with the defect in HR. Together these data suggest that phosphorylation of MRE11 plays a key role in determining extent of resection and that in the absence of this phosphorylation there is inefficient ATM-dependent phosphorylation of Exo1 which contributes to uncontrolled resection (Figure 7).

## 6. Conclusions 

ATM phosphorylates as many as 700 substrates as part of the response to DNA DSB. These substrates are part of a network of pathways that signal to the cell cycle, homologous recombination repair, transcription, regulation of spliceosomal activity, oxidative metabolism and protein synthesis. Three of these substrates Mre11, Rad50 and Nbs1 play a special and central role in this signalling. In the first instance this complex senses DNA DSB and recruits ATM to the sites of damage where it becomes activated to phosphorylate a myriad of substrates. The three members of the complex are amongst these substrates being upstream of a variety of events including cell cycle checkpoint control, DNA repair and cell survival. ATM phosphorylates Mre11 and Nbs1 at two sites and Rad50 at a single site (Table 2). Phosphorylation of Nbs1 appears to be necessary only for the S phase checkpoint but there are some reports that point to a wider involvement in repair and cell survival. Phosphorylation of Mre11 initiates signalling to control the extent of resection during HR repair and provides the signal for Mre11 to dissociate from DNA. On the other hand, a single phosphorylation site on Rad50, induced by DNA DSB, impacts on DNA repair, both HR and NHEJ, S phase cell cycle control and cell survival. It is also evident that agents that give rise to replicative stress induce Rad50 phosphorylation to assist in replication fork restart. Whether phosphorylation of the other two members of the complex is involved has not been determined. These phosphorylations do not affect the integrity of the complex *in vivo* so the downstream effects appear to be achieved in unison but with different outcomes dependent on the specific phosphorylation. In the case of Rad50 and Nbs1 phosphorylations, the downstream effects appear to be mediated through phosphorylation of SMC1, although not to an equivalent extent, since Nbs1 phosphorylation only controls the S phase checkpoint whereas phosphorylation of Rad50 impacts on several cellular processes. This may be due to signalling through as yet unidentified intermediate substrates of ATM. How this is achieved is also unclear but may rely on structural features of the complex. Phosphorylation of Mre11 on the other hand is directed to controlling extent of resection in HR. These data demonstrate the special position of the MRN complex in signalling *vis-à-vis* ATM. At this stage it is unclear how phosphorylation affects the structure of the MR complex. However, based on a schematic model for MRN obtained from structural analysis of bacterial and archaea MR complexes it exists in open and closed states. The closed state is associated with higher affinity for DNA ends and DNA tethering and the open state is required for Mre11 nuclease activity and promotion of resection [45]. Thus phosphorylation of Rad50, close to the zinc hook, might favour the closed state and Mre11 phosphorylation the open state compatible with resection. Use of phosphomimetic forms may assist in addressing this using *in vitro* systems.

**Table 2:** Different functional role of MRN complex protein phosphorylations

Member of complex	Number of sites	Consensus sequence	Phosphorylation Site	DNA damaging agent	Functional role
Nbs1	2	SQ	S278, S343	Irradiation	S-phase checkpoint
Mre11	2	SQ	S676, S678	Irradiation; Camptothecin; Etoposide	HDR repair (extent of resection); Survival
Rad50	1	SQ	S635	Irradiation; Ultraviolet; Hydroxyurea	DNA repair (HR and NHEJ); Survival; S-phase checkpoint
